# Bone mineral density changes among people living with HIV who have started with TDF-containing regimen: A five-year prospective study

**DOI:** 10.1371/journal.pone.0230368

**Published:** 2020-03-25

**Authors:** Win Min Han, Lalita Wattanachanya, Tanakorn Apornpong, Jureeporn Jantrapakde, Anchalee Avihingsanon, Stephen J. Kerr, Nipat Teeratakulpisarn, Tanate Jadwattanakul, Tawatchai Chaiwatanarat, Patinut Buranasupkajorn, Reshmie Ramautarsing, Nittaya Phanuphak, Sarat Sunthornyothin, Kiat Ruxrungtham, Praphan Phanuphak, TNT 003.1 study team

**Affiliations:** 1 The HIV Netherlands Australia Thailand Research Collaboration (HIV-NAT), Thai Red Cross AIDS Research Centre, Bangkok, Thailand; 2 Division of Endocrinology and Metabolism, Department of Medicine, and Hormonal and Metabolic Disorders Research Unit, Faculty of Medicine, Chulalongkorn University, Bangkok, Thailand; 3 Excellence Center for Diabetes, Hormone, and Metabolism, King Chulalongkorn, Memorial Hospital, Bangkok, Thailand; 4 Thai Red Cross AIDS Research Centre, Bangkok, Thailand; 5 Tuberculosis Research Unit, Faculty of Medicine, Chulalongkorn University, Bangkok, Thailand; 6 Department of Medicine, Faculty of Medicine, Chulalongkorn University, Bangkok, Thailand; 7 Biostatistics Excellence Centre, Faculty of Medicine, Chulalongkorn University, Bangkok, Thailand; 8 The Kirby Institute for Infection and Immunity in Society, University of New South Wales, Sydney, Australia; 9 Queen Savang Vadhana Memorial Hospital, Chonburi, Thailand; 10 Department of Radiology, Faculty of Medicine, Chulalongkorn University, Bangkok, Thailand; Institut Hospital del Mar d'Investigacions Mediques, SPAIN

## Abstract

There are limited data regarding long-term BMD changes over time among treatment-naïve people living with HIV (PLHIV) after initiating combined antiretroviral therapy (cART) in Asia. We aimed to study bone mineral density (BMD) changes among treatment-naïve PLHIV started treatment with tenofovir disoproxil fumarate (TDF)- or non-TDF-containing regimen and HIV-uninfected controls in an Asian setting. The study was a five-year prospective study. BMD at lumbar spine (LS) (L1 to L4), total hip (TH), and femoral neck (FN) were measured by dual energy X-ray absorptiometry (DEXA) scans at baseline, months 12, 24 and 60. Multivariate logistic regression models were used to explore factors associated with mean BMD ≥5% reduction after 5 years of cART. A total of 106 PLHIV (75 and 31 started TDF- and non-TDF-containing regimen, respectively) and 66 HIV-uninfected individuals were enrolled. The mean percent changes of BMD were significantly different longitudinally between TDF and non-TDF users (p<0.001 for LS, p = 0.006 for TH and p = 0.02 for FN). HIV-positive status and on TDF-containing regimen was independently associated with BMD loss ≥5% at month 60 (adjusted odds ratio [aOR] 7.0, 95% confidence interval [95%CI] 2.3–21.0, P = 0.001 for LS; aOR 4.9, 95%CI 1.7–14.3, P = 0.003 for TH and aOR 4.3, 95%CI 1.6–11.2, P = 0.003 for FN) compared to HIV-uninfected individuals. In a multivariate model for PLHIV only, TDF use (vs. non-TDF, P = 0.005) and pre-treatment CD4+ count <350 cells/mm^3^ (vs. ≥350 cells/mm^3^, P = 0.02) were independently associated with ≥5% BMD loss in TH at month 60. Treatment-naïve PLHIV initiating treatment with TDF-containing regimen have higher BMD loss in a Thai cohort. TDF use and low pre-treatment CD4 count were independently associated with BMD loss at month 60 at TH. Earlier treatment initiation and interventions to prevent bone loss could improve skeletal health among PLHIV.

**Clinicaltrials.gov**: NCT01634607

## Introduction

People living with HIV (PLHIV) have higher risks for decreased bone mineral density (BMD) and bone-related morbidities such as osteoporosis and fragility fractures compared to HIV-negative individuals [1–7]. Several factors including sex, body mass index (BMI), vitamin D level, hormonal effects, smoking, alcohol drinking and HIV-related factors such as advanced HIV disease and low baseline CD4+ cell count may contribute to BMD loss among PLHIV [8–12]. Additionally, HIV-infection induced immune activation and chronic inflammation may alter bone formation activities by negatively affecting osteoclasts and osteoblasts functions [13–16]. Those activities can be measured by using bone resorption and bone formation markers such as c-telopeptide crosslink of type 1 collagen (CTX) and procollagen type 1 N-terminal propeptide (P1NP). We recently reported that the levels of serum P1NP and osteocalcin (OC) were significantly lower in treatment-naïve PLHIV (especially among those with low pre-treatment CD4 cell count) than those without HIV infection in this Asian cohort [17].

Despite virological suppression with combination antiretroviral therapy (cART), treatment-associated adverse effects are still common among PLHIV in low-middle income settings where treatment options are limited. Certain antiretroviral drugs such as tenofovir disoproxil fumarate (TDF) and some protease inhibitors (PI) have been shown to be associated with reduced bone mineral density (BMD) in both treatment-naïve and treatment-experienced PLHIV [18–20]. Contribution of PI-based cART in reduced BMD among PLHIV may be mediated partly by toxic effects of concomitant TDF use. However, TDF-containing regimen is still the preferred first-line cART regimen in most of the resource-limited settings even when combined with dolutegravir [21]. It was suggested that TDF-containing regimen has been associated with BMD loss [22] but there is limited data on its long-term use. A study that compared the long-term BMD changes between PLHIV after TDF initiation and HIV-uninfected controls did not find HIV- or treatment-related factors associated with BMD loss after 24 months even though the rate of BMD decline was significantly greater in PLHIV in the first 24 months of TDF use [23]. Moreover, data on long-term BMD changes among virologically suppressed PLHIV in resource-limited settings are limited, especially from Asia. We aimed to study the long-term BMD changes prospectively using dual X-ray absorptiometry (DEXA) scan among treatment-naïve PLHIV who started their treatment with either TDF-containing or non-TDF-containing regimen and HIV-uninfected individuals.

## Methods

### Study population and settings

A prospective study was conducted to evaluate BMD changes among adult Thai treatment-naïve PLHIV after cART initiation with either TDF- or non-TDF-containing regimens from the TNT-HIV 003 bone sub-study. HIV-uninfected healthy participants were also enrolled from the TNT-HIV 003 cohort which was established in 2012 to study the morbidity and mortality among both healthy HIV-infected and HIV-uninfected individuals from Thai Red Cross AIDS Research Centre (TRC-ARC) in Bangkok, and the Queen Savang Vadhana Memorial Hospital in Chonburi, Thailand. The main inclusion criteria for the study were age ≥ 30 years and treatment-naive for PLHIV. The decision to treat patients with or without TDF was part of the standard of care. Both HIV-infected and HIV-uninfected participants who had history of metabolic bone diseases, wasting syndrome, malignancies, comorbidities such as diabetes mellitus and hypertension, advanced opportunistic infections and active use of immunosuppressive agents, steroids or anticonvulsants were excluded from the study. The duration of the study was 60 months after the last participant was enrolled. The study participants were recruited over the period between November 2010 and May 2014. The ART treatment decisions including the timing and types of cART were based on the relevant Thai HIV guidelines during the recruitment period.

### Study measurements

Demographic and clinical data including age, sex, BMI, waist circumference, smoking, alcohol drinking, substance use, fracture history and baseline HIV-related parameters such as CD4+ cell count, and HIV RNA levels were collected. Plasma was obtained and stored at -80°C until assayed. Estimated glomerular filtration rate (eGFR) was calculated by Chronic Kidney Disease Epidemiology Collaboration (CKD-EPI) equation. DEXA scans were also used to measure the body compositions such as total lean mass and body fat percentage.

DEXA scans were employed to measure BMD at the lumbar spine (LS) (L1 to L4), total hip (TH), and femoral neck (FN) by using a QDR 4500 bone densitometer (Hologic, Inc., Bedford, MA) at baseline, months 12, 24 and 60. BMD T-and Z-scores were analyzed using Asian population reference data supplied by the manufacturer. Serum calcium, phosphorus (ICMA; intact PTH assays Roche Elecsys^®^) and serum 25-hydroxyvitamin D (25(OH)D) levels (CLIA; DiaSorin LIAISON^®^) were measured at baseline. Baseline urine phosphate and creatinine levels were also assessed to calculate the fractional excretion of phosphate (FEPO4) by using the formula: [PO4 (Urine) * Creatinine (Serum)] / [PO4 (Serum) * Creatinine (Urine)] *100% [24, 25]. Additionally, bone turnover markers, P1NP, OC and CTX, were measured only at baseline by using a chemiluminescence immunoassay (Roche Diagnostics, Mannheim, Germany).

### Definitions of the study endpoints

The prevalence of clinical status of reduced BMD was measured by osteoporosis and osteopenia events among study participants. Osteoporosis and osteopenia were defined as a T-score ≤ -2.5 standard deviation (SD) and between -1 and -2.5 SD below the young adult mean value [26], respectively. We also reported Z-score from the classification system of the International Society for Clinical Densitometry (ISCD), which defines a Z-score ≤ -2.0 SD as “below the expected range for age”. The study’s main outcome for the modelling analyses was defined as ≥ 5% reduction in the mean BMD (grams per square centimeter, g/cm^2^) after 60 months of cART at each anatomical site.

### Statistical analysis

Demographic data and baseline HIV-related parameters, levels of bone markers, BMD at different sites, calcium and vitamin D status were described descriptively as median (IQR), mean (SD), or number (percentage). Comparisons between the study groups (HIV-infected vs. HIV-uninfected and TDF-containing regimen vs. non-TDF-containing regimen) were done by using t-test or Mann-Whitney U tests for continuous variables and Chi-square or Fisher’s Exact tests for categorical variables. Relative BMD changes of the early phase (month 0 to month 24) and late phase (month 0 to month 60) of cART initiation were also compared for each anatomical site. Univariate and multivariate logistic regression models were employed to evaluate factors associated with reduced BMD ≥ 5% at month 60 (compared to baseline) at each site. The scans from PLHIV on TDF-containing regimen were compared to non-TDF containing regimen. Age, sex, BMI and all other variables with p-values < 0.15 were adjusted in multivariate regression models. Separate models were also employed to evaluate HIV-related variables associated with reduction of mean BMD ≥ 5% at month 60 among PLHIV only. A p-value less than 0.05 was considered significant. Sensitivity analyses were done by decreasing the cut off of BMD reduction at month 60 from ≥ 5% to ≥ 3% for all three anatomical sites. All analyses were done using STATA version 15.1 (STATA Corp LLC, College Station, TX).

### Ethical consideration

The study was reviewed and approved by the Institutional Review Board of the Faculty of Medicine, Chulalongkorn University, Bangkok, Thailand. All HIV-infected and HIV-uninfected participants in the study provided written informed consents.

## Results

### Participants’ characteristics

A total of 106 PLHIV (75 started with TDF-containing regimen and 31 started with non-TDF-containing regimen) and 66 HIV-uninfected individuals were included in the study analyses. Demographics and baseline characteristics of participants were stratified by either HIV status or by the use of TDF as displayed in Table 1. Median age of all participants was 38.3 (interquartile range [IQR] 35.3–44.0) years. In our study, PLHIV were relatively younger than HIV-uninfected individuals (37.2 vs. 41.1 years, P< 0.001). Both PLHIV and HIV-uninfected individuals had comparable body compositions which were determined by total lean mass and body fat percentages from DEXA scans. The number of participants still in the study at month 60 for the TDF group was 53/101 (52%), and for the non-TDF group, it was 19/49 (39%). Therefore, non-TDF group had a higher rate of loss to follow-up (LTFU) at month 60 (30/49, 61%).

**Table 1:** Baseline characteristics of the study participants.

Baseline covariates	Total (n = 172)	HIV-uninfected (n = 66)	HIV-infected (n = 106)	P-value	TDF-containing regimen (n = 75)	Non-TDF containing regimen (n = 31)	P-value
**Sex**				0.14			0.9
Male	88 (51.16)	29 (43.94)	59 (55.66)		42 (56.00)	17 (54.84)	
Female	84 (48.84)	37 (56.06)	47 (44.34)		33 (44.00)	14 (45.16)	
**Age**, median (IQR)	38.3 (35.3–44.0)	41.1 (37.2–46.3)	37.2 (34.5–41.5)	0.0001	37.0 (34.5–41.9)	38.2 (34.1–40.9)	0.87
**BMI (kg/m**^**2**^**)**	22.7 (21.0–24.9)	23.8 (21.2–25.8)	22.5 (20.9–24.7)	0.11	22.5 (21.0–24.4)	22.5 (20.8–25.9)	0.56
Abnormal waist*, n (%)	62 (36.05)	31 (46.97)	31 (29.25)	0.02	18 (24.00)	13 (41.94)	0.07
**Dyslipidemia**	69/171 (40.4)	41/66 (62.1)	28/105 (26.7)	<0.001	20/74 (27)	8/31 (25.8)	0.90
**Fracture history**	18 (23.1)	5 (27.8)	13 (21.7)	0.75	9/42 (21.4)	4/18 (22.2)	1.00
**eGFR (mL/min/1.73m**^**2**^**)**	89.4 (75.8–105.4)	85.3 (75.2–108.1)	92.6 (77.4–104.2)	0.83	88.2 (77.5–103.3)	96.1 (75.0–108.1)	0.52
**25 (OH) D (ng/mL)**	28.9 (22.9–34.3)	24.1 (18.6–29.7)	31.2 (26.8–36.5)	<0.001	31.2 (26.4–36.2)	30.6 (27.8–37.7)	0.76
**25 (OH) D, ng/mL, n (%)**				<0.001			0.20
<20 (deficiency)	26 (16.1)	20 (30.8)	6 (6.2)		6 (8.6)	0 (0)	
20–30 (insufficiency)	68 (42.0)	31 (47.7)	37 (38.1)		24 (34.3)	13 (48.2)	
30–100 (sufficiency)	68 (42.0)	14 (21.5)	54 (55.7)		40 (57.1)	14 (51.9)	
**HIV-related variables**							
**Baseline CD4 count** (cells/mm^3^)			274 (173–379)		285 (164–412)	253 (175–312)	0.22
**Baseline HIV-RNA level** (log_10_ copies/mL)			4.8 (4.3–5.2)		4.8 (4.2–5.2)	4.7 (4.4–5.3)	0.61
**CDC staging**							0.51
A			76 (71.70)		56 (74.67)	20 (64.52)	
B			27 (25.47)		17 (22.67)	10 (32.26)	
C			3 (2.83)		2 (2.67)	1 (3.23)	
**Protease inhibitors use**			4 (3.77)		1 (1.33)	3 (9.68)	0.07
**Bone markers**							
Serum calcium, mg/dL	9.5 (9.3–9.7)	9.6 (9.3–9.8)	9.4 (9.1–9.7)	0.005	9.4 (9.1–9.7),	9.4 (9.2–9.7)	0.55
Serum phosphate, mg/dL	3.8 (3.5–4.2)	3.9 (3.6–4.2)	3.8 (3.5–4.2)	0.71	3.8 (3.5–4.2)	3.7 (3.5–4.2)	0.49
OC, l ng/mL	13.2 (10.0–17.9)	15.9 (13.1–20.2)	11.0 (9.0–15.2)	<0.001	11.2 (9.7–14.9)	10.5 (8.7–17.4)	0.58
CTX, ng/mL	0.3 (0.2–0.4)	0.3 (0.2–0.4)	0.2 (0.1–0.3)	0.0007	0.2 (0.1–0.3)	0.2 (0.1–0.3)	0.68
P1NP, ng/mL	38.2 (29.8–50.9)	44.7 (36.6–55.1)	33.1 (27.4–42.6)	0.0001	32.9 (27.5–41.7)	34.6 (26.0–48.7)	0.57
**BMD at lumbar spine**							
BMD (g/cm^2^)	0.98 (0.12)	0.97 (0.12)	0.99 (0.12)	0.22	1.00 (0.12)	0.99 (0.11)	0.68
T-score, mean (SD)	-0.26 (1.02)	-0.37 (1.04)	-0.19 (1.00)	0.27	-0.18 (1.04)	-0.24 (0.92)	0.76
Z-score, mean (SD)	-0.02 (0.90)	-0.05 (0.89)	0.00 (0.91)	0.70	0.03 (0.93)	-0.06 (0.87)	0.66
**BMD at hip**							
BMD (g/cm^2^)	0.92 (0.13)	0.90 (0.12)	0.93 (0.13)	0.13	0.92 (0.13)	0.95 (0.12)	0.37
T-score, mean (SD)	0.22 (1.02)	0.11 (0.95)	0.29 (1.06)	0.24	0.23 (1.08)	0.45 (1.01)	0.32
Z-score, mean (SD)	0.37 (1.01)	0.30 (0.94)	0.41 (1.05)	0.48	0.35 (1.06)	0.56 (1.02)	0.35
**BMD at femoral neck**							
BMD (g/cm^2^)	0.79 (0.12)	0.77 (0.11)	0.80 (0.13)	0.17	0.79 (0.13)	0.82 (0.13)	0.35
T-score, mean (SD)	-0.31 (1.04)	-0.46 (0.94)	-0.22 (1.09)	0.18	-0.28 (1.09)	-0.05 (1.09)	0.31
Z-score, mean (SD)	0.14 (1.00)	0.05 (0.89)	0.20 (1.07)	0.48	0.14 (1.07)	0.35 (1.06)	0.35

**Abbreviations**: BMI, body mass index; eGFR, estimated glomerular filtration rate; 25(OH)D, 25-hydroxyvitamin D; OC, osteocalcin; CTX, c-telopeptide crosslink of type 1 collagen; P1NP, procollagen type 1 N-terminal propeptide.

*Abnormal waist was defined as having waist circumference ≥ 80 cm in female and ≥ 90 cm in male. Dyslipidemia was defined as total cholesterol ≥200 mg/dL.

The two groups, TDF and non-TDF users, had similar median age at study entry (37.0 vs. 38.2 years, P = 0.8). At baseline, the participants from both groups did not have a proteinuria event. Sixty-eight percent of the participants started treatment with TDF, lamivudine and emtricitabine or efavirenz (TDF/3TC(or)FTC/EFV) as their initial regimen. PI use was only 3.77% among the study participants. There were no differences in BMI at baseline between the two treatment groups (P = 0.56). All participants from different treatment groups had similar total body fat percentages (27 vs. 25%, P = 0.9) and total lean mass (41.5 vs. 42.2 kg, P = 0.8) at baseline.

Baseline levels of serum calcium, phosphate, 25(OH)D, and bone turnover markers are presented in Table 1. There were no significant differences in baseline levels of 25(OH)D (p = 0.76), OC (p = 0.58), CTX (p = 0.68) and P1NP (p = 0.57) between those starting treatment with TDF- or non-TDF-containing regimens. The prevalence of abnormal vitamin D level (insufficiency or deficiency) at baseline was similar in both groups. There were also no differences in serum calcium and phosphate levels between the groups.

However, ARV-naïve PLHIV had lower pre-treatment bone turnover markers (OC, CTX, and P1NP) than HIV-uninfected controls at baseline. Of note, PLHIV had higher vitamin D level than HIV-uninfected individuals at baseline (24.1 IQR, 18.6–29.7 vs. 31.2 IQR, 26.8–36.5 ng/mL, p<0.001). Also, the baseline BMD and mean Z- and T-scores were similar among the two treatment groups (Table 1).

### Percentage of mean BMD changes from baseline to months 12, 24 and 60 in PLHIV on TDF-containing versus non-TDF-containing regimen

**Fig 1:**
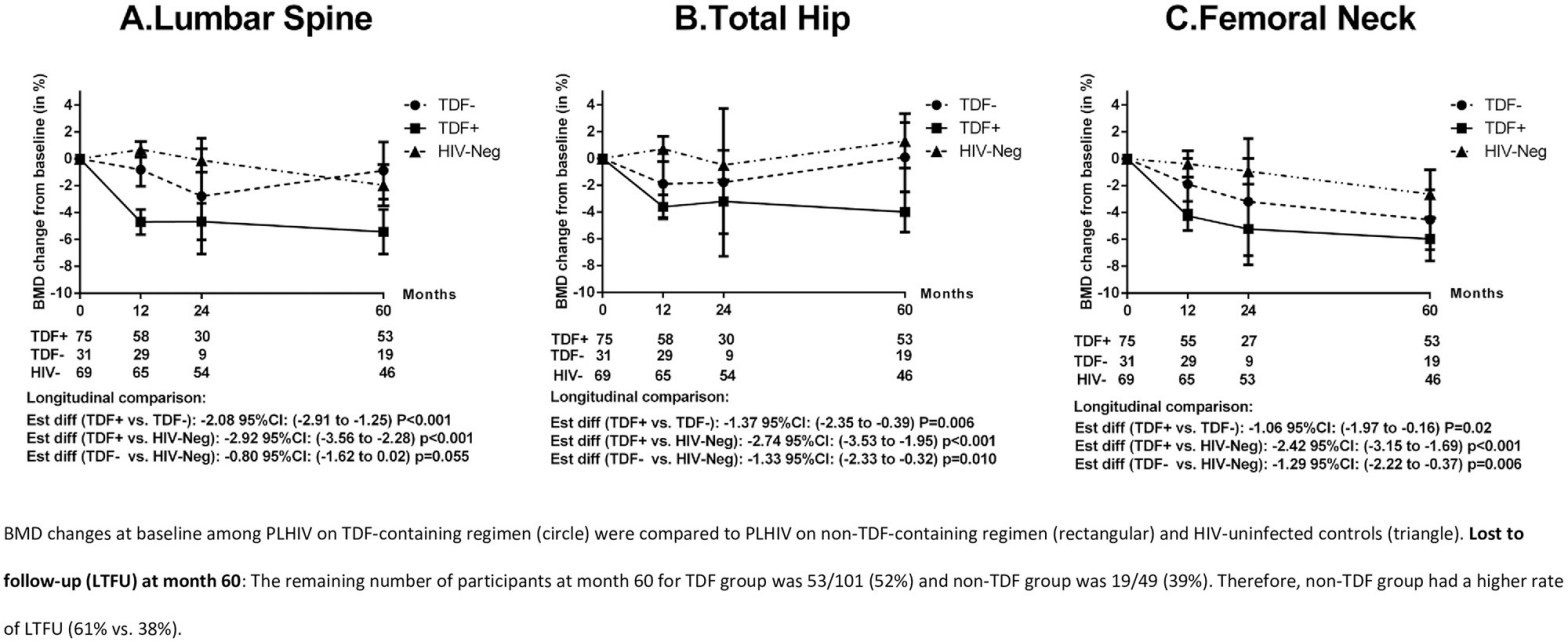
Percent change of BMD at baseline, months 12, 24 and 60.

The mean percentage changes of BMD at different anatomical sites at months 12, 24 and 60 are shown in Fig 1. The mean percentage changes of BMD for TDF and non-TDF users in LS (Fig 1A) were -4.7% versus. -0.8% at month 12, -4.7% vs. -2.8% at month 24, and -5.4% vs. -0.9% at month 60, respectively (p<0.001). For TH (Fig 1B), percentage changes in BMD for TDF and non-TDF groups were -3.6% vs. -1.9% at month 12, -3.2% vs. -1.8% at month 24 and -4% vs. 0.1% at month 60, respectively (p = 0.006). The changes were also significant between the two groups for FN over the study period, p = 0.02 (Fig 1C). For longitudinal comparison over 60 months of treatment, TDF users had significantly higher mean BMD loss of -2.08% (95% CI, -2.91 to -1.25), p<0.001) in LS, -1.37% (95% CI, -2.35 to -0.39, p = 0.006) in TH, and -1.06 (95% CI, -1.97 to -0.16), p = 0.02) in FN, than non-TDF users.

We also compared the BMD changes from baseline to month 12 versus months 12 to 24 (Table 2). Changes in BMD from baseline to month 12 among TDF users were significantly higher than the changes in months 12 to 24 [-3.9% (95% CI, -5.7 to -1.4) vs. -0.1% (95% CI, -2.0 to 0.6), p<0.001 for LS, -4.2% (-6.9 to -2.4) vs. -0.4% (-3.0 to 1.2), p = 0.002 for TH and -4.6% (-7.9 to -2.7) vs. 0% (-2.9 to 2.1), p = 0.017 for FN]. Of note, the BMD changes among TDF users from baseline to month 24 were statistically higher than the changes from months 24 to 60 at LS, (-3.58% vs. 0%, p = 0.014), TH (-5.5% vs. -0.2%, p = 0.008) and FN (-6.1 vs. -0.5%, p = 0.002) (Table 2).

**Table 2:** Comparison of relative BMD changes between the early phase (month 0 to month 24) and late phase (month 0 to month 60) of treatment.

Relative BMD changes	Month 0 –month 12	Month 0 –month 24	P-value	Month 0 –month 24	Month 0 –month 48	P-value
	*Median (95% CI)*	*Median (95% CI)*		*Median (95% CI)*	*Median (95% CI)*	
**Lumbar Spine**						
TDF	-3.9 (-5.7 to -1.4)	-0.1 (-2 to 0.6)	**<0.001**	-3.6 (-6.4 to -1.0)	0.0 (-2.1 to 2.8)	**0.014**
Non-TDF	-1.3 (-3.5 to -0.7)	-0.1 (-2.1 to 2.2)	0.37	-0.9 (-2.2 to 0.89)	-0.2 (-2.0 to 1.6)	0.395
**Total Hip**						
TDF	-4.2 (-6.9 to -2.4)	-0.4 (-3.0 to 1.2)	**0.002**	-5.5 (-7.7 to -3.5)	-0.2 (-4.0 to 3.3)	**0.008**
Non-TDF	-2.7 (-3.5 to 0.3)	0.1 (-0.6 to 1.5)	0.63	-0.2 (-3.0 to 6.6)	0.5 (-6.5 to 2.6)	0.65
**Femoral Neck**						
TDF	-4.6 (-7.9 to -2.7)	0.00 (-2.9 to 2.1)	**0.017**	-6.1 (-8.9 to -3.7)	-0.5 (-2.5 to 1.8)	**0.002**
Non-TDF	-3.8 (-8.4 to 2.0)	1.4 (-2.0 to 2.4)	0.08	-2.5 (-6.0 to -0.9)	-2.8 (-3.6 to -1.2)	0.45

### Prevalence of combined osteopenia and osteoporosis at baseline and month 60

**Fig 2:**
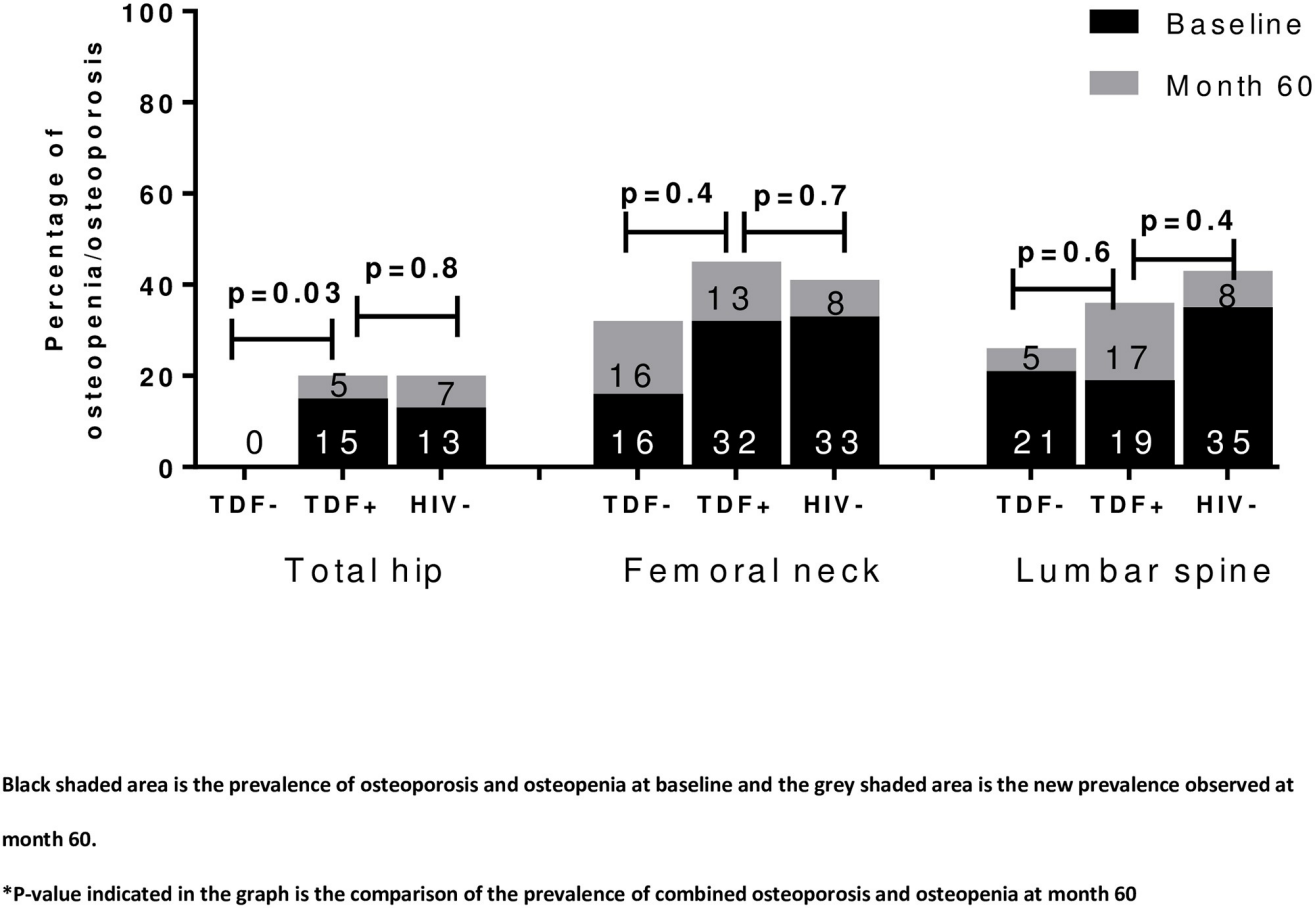
Prevalence of combined osteopenia and osteoporosis among PLHIV on either TDF-containing or non-TDF-containing regimen at month 0 and 60.

Overall, there were some participants who had osteoporosis in our study over the follow-up years. At baseline, the prevalence of combined osteoporosis and osteopenia in TH, FN and LS was 0%, 16% and 21% among treatment-naïve PLHIV who initiated treatment with non-TDF-containing regimen and 15%, 32% and 19% among those who started treatment with TDF-containing regimen, respectively. The prevalence of combined osteoporosis or osteopenia was then increased to 0%, 32% and 26% in in TH, FN and LS among non-TDF users and 20%, 55% and 36% among TDF users after 60 months of cART (Fig 2). There were no significant differences in the prevalence of combined osteoporosis and osteopenia between TDF users and HIV-uninfected individuals at each site at 60 months. In addition, there were no differences in the proportions of participants with Z-scores ≤ −2.0 SD between the two treatment arms of each anatomical site at any time point.

### Factors associated with ≥ 5% BMD loss at month 60 in both HIV-infected and HIV-uninfected individuals

The prevalence of BMD loss ≥ 5% among both HIV-infected and HIV-uninfected participants was 35%, 28% and 42% at in TH, FN and LS, respectively. The logistic regression models were employed to evaluate the variables associated with the outcomes of ≥ 5% reduction in BMD from baseline to month 60. In the multivariate models, when we included both PLHIV and HIV-uninfected individuals, we found that the HIV-positive status and TDF-containing regimen were independently associated with ≥ 5% decrease in BMD at month 60 for all sites (adjusted odds ratio [aOR] 7.0, 95% confidence interval [95% CI] 2.3–21.0, P = 0.001 for LS; aOR 4.9, 95% CI 1.7–14.3, P = 0.003 for TH and; aOR 4.3, 95% CI 1.6–11.2, P = 0.003 for FN) compared to HIV-uninfected individuals after adjusting for age, sex and BMI (Table 3). The associations remained significant after controlling for bone turnover markers (CTX and P1NP) and FEPO4 at baseline in the multivariate analyses. In addition, women were more likely to have BMD loss at LS (aOR 4.1, 95% CI 1.7–10.2, P = 0.02) compared to their counterparts. From the model analyses, we did not find the associations of bone turnover markers, use of PI-based cART, 25(OH)D levels and other traditional risk factors such as BMI, waist circumference, total lean or total fat mass, smoking and alcohol consumption, with ≥ 5% BMD loss at month 60 at the different anatomical sites.

**Table 3:** Multivariate analyses for ≥5% BMD loss at month 60 at the lumbar spine, total hip and femoral neck.

**Baseline variates for both PLHIV and HIV-uninfected participants**	BMD ≥5% reduction at lumbar spine* **(prevalence, 35%)**		BMD ≥5% reduction at total hip* **(prevalence, 28%)**		BMD ≥5% reduction at femoral neck* **(prevalence, 42%)**	
	**aOR (95% CI)**	**P-value**	**aOR (95% CI)**	**P-value**	**aOR (95% CI)**	**P-value**
**Age** (vs. <40 years)						
≥40	1.5 (0.6–3.7)	0.4	1.2 (0.5–3.1)	0.7	1.7 (0.7–3.9)	0.2
**Female** (vs. male)	**4.1 (1.7–10.2)**	**0.02**	1.9 (0.8–4.6)	0.2	1.2 (0.5–2.6)	0.6
**BMI** (vs. <25 kg/m^2^)						
≥25	1.7 (0.6–4.5)	0.3	1.4 (0.5–3.8)	0.6	2.2 (0.9–5.5)	0.1
**HIV status** (vs. HIV negative)						
HIV started with TDF	**7.0 (2.3–21.0)**	**0.001**	**4.9 (1.7–14.3)**	**0.003**	**4.3 (1.6–11.2)**	**0.003**
HIV started with non-TDF	2.1 (0.5–8.1)	0.3	0.7 (0.1–3.7)	0.7	1.7 (0.5–5.9)	0.4
**Baseline variates for PLHIV only**	BMD ≥5% reduction at lumbar spine** **(prevalence, 43%)**		BMD ≥5% reduction at total hip** **(prevalence, 35%)**		BMD ≥5% reduction at femoral neck** **(prevalence, 50%)**	
	**aOR (95% CI)**	**P-value**	**aOR (95% CI)**	**P-value**	**aOR (95% CI)**	**P-value**
**Age** (vs. <40 years)						
≥40	1.2 (0.4–3.6)	0.8	1.0 (0.3–3.4)	1.0	2.4 (0.8–7.2)	0.1
**Female** (vs. male)	**3.0 (1.0–8.8)**	**0.05**	2.1 (0.7–6.7)	0.2	1.1 (0.4–3.2)	0.8
**BMI** (vs. <25 kg/m^2^)						
≥25	3.2 (0.8–13.1)	0.1	3.1 (0.7–14.5)	0.1	2.9 (0.7–11.3)	0.1
**cART regimen** (vs. non-TDF)						
TDF	3.1 (0.9–11.1)	0.1	**11.7 (2.1–64)**	**0.005**	2.8 (0.8–9.2)	0.1
**Baseline CD4+ cell count** (vs. ≥350 cells/mm3)						
< 350 cells/mm3	1.9 (0.5–7.1)	0.3	**7.1 (1.3–38.8)**	**0.02**	2.3 (0.7–7.9)	0.2
**Baseline HIV RNA level** (log_10_ copies/mL)	2.1 (1.0–4.5)	0.06	0.8 (0.4–1.9)	0.7	1.2 (0.6–2.5)	0.5

* Multivariate models with both PLHIV and HIV-uninfected controls were adjusted for age, sex, BMI and HIV status.

** Multivariate models with only PLHIV that were adjusted for age, sex, BMI, cART regimen (TDF vs. non-TDF), baseline CD4 count and baseline HIV-RNA level.

### Factors associated with ≥ 5% BMD loss at month 60 among HIV-infected individuals

The prevalence of BMD loss ≥ 5% among TDF users was higher than non-TDF users (49% vs. 26%, p = 0.086 in LS, 43% vs. 11%, p = 0.01 in TH and 55% vs. 37%, p = 0.18 in FN, respectively). In separate multivariate models for PLHIV only, we found that TDF use (vs. non-TDF, aOR 11.7, 95% CI 2.1–64, P = 0.005) and baseline CD4+ cell count < 350 cells/mm^3^ (vs. ≥ 350 cells/mm^3^, aOR 7.1, 95% CI 1.3–38.8, P = 0.02) were independently associated with ≥ 5% BMD loss (Table 3) at TH but not at the other sites at month 60 after adjusting for age, BMI, baseline CD4 count, HIV RNA level and other significant cofounders from the univariate model. However, women were more likely to have BMD loss than men at LS (aOR 3.0, 95% CI 1.0–8.8, P = 0.05). In other models, we did not find the associations of HIV RNA level, CDC staging, body fat and lean mass, serum calcium, 25(OH)D levels and bone turnover markers, with BMD loss at month 60.

### Sensitivity analysis

A sensitivity analysis using month 60 BMD loss cut off as ≥ 3% was performed to test the robustness of our findings. The significant associations of HIV-infected status and TDF use remained unchanged at all three anatomical sites. However, the association of baseline CD4 cell count and ≥ 3% BMD reduction at TH was lost in the sensitivity analysis.

## Discussion

There are limited data regarding long-term BMD changes over time among treatment-naïve PLHIV after initiating cART in Asia. In this prospective study, we determined the long-term BMD changes among HIV-uninfected individuals and treatment-naïve PLHIV initiating either TDF- or non-TDF-containing regimens. We found that TDF users were likely to have greater BMD loss than non-TDF users at month 60. Additionally, our findings suggest that initial cART regimen containing TDF had the greatest BMD loss in the first 12 months of its use. From the multivariate models, including both PLHIV and negative controls, we found that those started with TDF-containing regimen (compared to HIV-uninfected participants) was independently associated with ≥5% BMD loss at month 60 at all three anatomical sites studied. Among the models in this study, only PLHIV who started treatment with TDF-containing regimen and the female sex were independently associated with BMD loss at month 60 at LS (but not at TH or FN) after potential confounders were controlled.

The results of BMD changes of our study were in line with previous reports that the BMD loss among PLHIV was associated with TDF use and low pre-treatment CD4+ cell count [12, 27, 28]. Previous evidences showed that among treatment naïve HIV participants whose initial regimen was TDF-based regimen had nearly 1–3% BMD loss at 12 to 36 months after starting treatment compared to those who had non-TDF containing regimen [18, 20, 27, 29–31]. Our data supported those previous evidences that BMD loss was higher in the first 12 months and stabilized after 24 months of using TDF-containing cART as the initial regimen. However, few studies have evaluated long-term BMD changes with TDF use. In our cohort, we found that PLHIV on TDF-containing regimen was an independent risk factor for BMD loss of ≥ 5% from baseline to 60 months at total hip after potential confounders were controlled in the analyses. Furthermore, we also found that there was an independent association of low pre-treatment CD4+ cell count with BMD loss at total hip. The association of low baseline CD4+ cell count and BMD loss suggests that the immune system could have potential roles in the changes of the metabolism of the skeletal tissues.

In our study, women living with HIV had higher risks for BMD loss at month 60 at the LS (but not at TH or FN). Given that women have higher skeletal fracture risks than men, this is an important implication that HIV-infected women should be screened for osteoporosis or osteopenia regularly. Interventions such as treatment with bisphosphate and switching to other bone-friendly cART regimen with less BMD toxicities should be considered for those found to have osteoporosis and osteopenia.

Furthermore, previous studies have explored the association of body weight with BMD loss among PLHIV [7, 32]. However, we did not observe the associations of BMI or waist circumference with BMD loss at month 60 at any anatomical sites. In addition, our study did not find the association of HIV RNA level and BMD loss. This could possibly be explained by the reason that our study participants had successful treatment or that the effects of ARV (for instance, TDF) overwhelmed the viremia effect. We also failed to observe the relationship of advanced HIV disease with BMD loss. It might be because our participants were relatively healthy at cART initiation since individuals with wasting syndrome or opportunistic infections were excluded at the screening visit.

HIV-induced chronic immune activation could affect bone metabolism and hence increase bone loss. In this study, we did not find associations of baseline urinary phosphate wasting and bone turnover markers with BMD change in the multivariate models. However, several reports have suggested the predictive role of baseline bone turnover markers for BMD loss among TDF users [16] or BMD gain after switching from TDF [33]. For example, one study evaluated BMD changes after switching from TDF to tenofovir alafenamide (TAF) among PLHIV with low BMD; TDF-treated individuals with high baseline PN1P levels who switched to TAF had increased BMD after 24 months on TAF [33]. Moreover, a number of studies have suggested that there is a relationship between BMD loss and low serum 25(OH)D levels. In this study, we did not find such an association. It could be explained by the fact that most of the study participants were healthy and had low rate of vitamin D deficiency at baseline in our cohort. However, PLHIV started with TDF-containing regimen had greater BMD loss than uninfected individuals although serum 25(OH)D levels were higher among all PLHIV at baseline. The prevalence of osteoporosis was low. A possible explanation for this is that our study participants had few traditional risk factors for BMD loss and the young median age at cART initiation. Another plausible explanation is that the study used the outcome as BMD loss at month 60 with the cut off of 5% and this could overestimate bone loss in the cohort. In a systematic review of cART in the early years showed that treatment-naïve individuals on TDF containing regimen had 1–3% BMD loss at 12 to 24 months. When a 3% cut off was used for the sensitivity analysis, we found a significant association between TDF use and BMD loss at month 60.

Several limitations of our study should be acknowledged. First, there were high rates of LTFU among the study participants, especially among PLHIV from the non-TDF group. Therefore, the risk factors identified in our results should be interpreted cautiously and further studies with a larger sample size may be needed to confirm these findings. Second, the measurements of the bone markers were only done at baseline visits. Third, the treatment arm (TDF or non-TDF) was not randomly assigned to treatment-naïve HIV-infected participants, and hence, the selection bias could not be totally excluded. However, all HIV-infected and healthy HIV-uninfected participants enrolled in the study did not have comorbidities such as diabetes, hypertension and bone metabolic diseases. Moreover, our study is the first Asian cohort that assessed the BMD loss in treatment-naïve HIV-infected individuals using TDF- or non TDF containing regimens over a 5-years period. DEXA scans were done at baseline, months 12, 24 and 60. These data were analyzed and compared to the results from HIV-uninfected controls which had equal numbers of men and women as the PLHIV group.

## Conclusion

We found that treatment-naïve HIV-infected individuals who started their treatment with TDF-containing regimen continued to have BMD loss at 5 years even though the rate was slower after 24 months of treatment. Compared with HIV-uninfected individuals and PLHIV on non-TDF-containing regimen, TDF users were more likely to have BMD loss at all three anatomical sites. In addition, lower pre-treatment CD4 count was associated with BMD loss at month 60 at total hip. Therefore, early HIV diagnosis and rapid cART initiation or interventions to prevent BMD loss may improve the bone health among PLHIV with long-term virological suppression.

## Supporting information

S1 Checklist Strobe statement—Checklist of items that should be included in reports of *cohort studies*. (DOC)
