# Supplementary material for: Bone mineral density changes among people living with HIV who have started with TDF-containing regimen: A five-year prospective study
Source: PLoS One. 2020 Mar 25;15(3):e0230368. doi: 10.1371/journal.pone.0230368 (PMC7094833; doi:10.1371/journal.pone.0230368)
Supplement: S1 Checklist — (DOC) [file pone.0230368.s001.doc]

STROBE Statement—Checklist of items that should be included in reports of ***cohort studies***

|  | Item No | Recommendation |
| --- | --- | --- |
| **Title and abstract** | 1 | (*a*) Indicate the study’s design with a commonly used term in the title or the abstract  Title: “Bone Mineral Density Changes among People Living with HIV Who Have Started with TDF-containing regimen: A Five-year Prospective Study.” |
|  |  | (*b*) Provide in the abstract an informative and balanced summary of what was done and what was found  Provided in abstract ‘Methods and Findings’ section. |
| Introduction | | |
| Background/rationale | 2 | Explain the scientific background and rationale for the investigation being reported  Explained in Introduction paragraphs 1-2 |
| Objectives | 3 | State specific objectives, including any prespecified hypotheses  Stated in Introduction paragraph 2 (last sentence) |
| Methods | | |
| Study design | 4 | Present key elements of study design early in the paper  Presented in Methods paragraph 1 |
| Setting | 5 | Describe the setting, locations, and relevant dates, including periods of recruitment, exposure, follow-up, and data collection  Described in Introduction last paragraph and Methods paragraph 1 with the sub-heading “Study population and settings” |
| Participants | 6 | (*a*) Give the eligibility criteria, and the sources and methods of selection of participants. Describe methods of follow-up  Described in Methods paragraph 1 with the sub-heading “Study population and settings” |
|  |  | (*b*) For matched studies, give matching criteria and number of exposed and unexposed  Not a matched study |
| Variables | 7 | Clearly define all outcomes, exposures, predictors, potential confounders, and effect modifiers. Give diagnostic criteria, if applicable  Defined in Methods paragraphs with sub-headings “Study measurements” and “Definitions of the study endpoints” |
| Data sources/ measurement | 8* | For each variable of interest, give sources of data and details of methods of assessment (measurement). Describe comparability of assessment methods if there is more than one group  Described in Methods “Definitions of the study endpoints” |
| Bias | 9 | Describe any efforts to address potential sources of bias  Sensitivity analyses described in Methods “Statistical analysis”. |
| Study size | 10 | Explain how the study size was arrived at  Described in Methods “Study population and settings” |
| Quantitative variables | 11 | Explain how quantitative variables were handled in the analyses. If applicable, describe which groupings were chosen and why  This is partially explained in the methods section under “Statistical analysis” sub-heading. Moreover, from statement of the results in Tables 1 and Table 3, it can be seen that quantitative variables were presented and analysed in descriptive as well as multivariable analysis as categorical variables. |
| Statistical methods | 12 | (*a*) Describe all statistical methods, including those used to control for confounding  Statistical methods are detailed in Methods “Statistical analysis” paragraph. |
|  |  | (*b*) Describe any methods used to examine subgroups and interactions  Methods to examine the subgroups of different anatomical sites |
|  |  | (*c*) Explain how missing data were addressed  Only the participants with at least one DEXA scan available were included in the study analysis. The study excluded 72 HIV-infected and 6 HIV-uninfected participants without BMD data. |
|  |  | (*d*) If applicable, explain how loss to follow-up was addressed  LTFU was a competing event accounted for in our modelling strategy. The limitation of high LTFU in our cohort was discussed in Discussion paragraph’s limitations. |
|  |  | (*e*) Describe any sensitivity analyses  Sensitivity analyses are described in Methods “Sensitivity analysis”. |
| Results | | |
| Participants | 13* | (a) Report numbers of individuals at each stage of study—eg numbers potentially eligible, examined for eligibility, confirmed eligible, included in the study, completing follow-up, and analysed  This was explained in Results paragraph “Participants’ characteristics” |
|  |  | (b) Give reasons for non-participation at each stage  Not applicable |
|  |  | (c) Consider use of a flow diagram  Not applicable |
| Descriptive data | 14* | (a) Give characteristics of study participants (eg demographic, clinical, social) and information on exposures and potential confounders  See Results Paragraph Participants’ characteristics and Table 1. |
|  |  | (b) Indicate number of participants with missing data for each variable of interest  The study excluded 72 HIV-infected and 6 HIV-uninfected participants without BMD outcomes. All the participants have the data for variables we included in the analyses. |
|  |  | (c) Summarise follow-up time (eg, average and total amount)  Described in Methods “Study population and settings” |
| Outcome data | 15* | Report numbers of outcome events or summary measures over time  This was stated in the results paragraph “Factors associated with ≥ 5% BMD loss at month 60 among HIV-infected individuals” and Table 3. |
| Main results | 16 | (*a*) Give unadjusted estimates and, if applicable, confounder-adjusted estimates and their precision (eg, 95% confidence interval). Make clear which confounders were adjusted for and why they were included  Results paragraph “Factors associated with ≥ 5% BMD loss at month 60 among HIV-infected individuals” and Tables 3. |
|  |  | (*b*) Report category boundaries when continuous variables were categorized  Results Tables 1 and 2. |
|  |  | (*c*) If relevant, consider translating estimates of relative risk into absolute risk for a meaningful time period  Not applicable |
| Other analyses | 17 | Report other analyses done—eg analyses of subgroups and interactions, and sensitivity analyses  Sensitivity analyses of with BMD reduction ≥ 3% were done and explained in the Results “Sensitivity analysis”. |
| Discussion | | |
| Key results | 18 | Summarise key results with reference to study objectives:  Summarized in Discussion paragraph 1. |
| Limitations | 19 | Discuss limitations of the study, taking into account sources of potential bias or imprecision. Discuss both direction and magnitude of any potential bias  Limitations are presented in Discussion paragraph 7. |
| Interpretation | 20 | Give a cautious overall interpretation of results considering objectives, limitations, multiplicity of analyses, results from similar studies, and other relevant evidence  Internal and external comparisons are given in Discussion paragraph 2, 3 and 4. Overall interpretation is given in Conclusion. |
| Generalisability | 21 | Discuss the generalisability (external validity) of the study results  This is discussed in the limitations section, Discussion - last paragraph |
| Other information | | |
| Funding | 22 | Give the source of funding and the role of the funders for the present study and, if applicable, for the original study on which the present article is based  See the Funding Statement entered at submission and in the manuscript |

*Give information separately for exposed and unexposed groups.

**Note:** An Explanation and Elaboration article discusses each checklist item and gives methodological background and published examples of transparent reporting. The STROBE checklist is best used in conjunction with this article (freely available on the Web sites of PLoS Medicine at http://www.plosmedicine.org/, Annals of Internal Medicine at http://www.annals.org/, and Epidemiology at http://www.epidem.com/). Information on the STROBE Initiative is available at http://www.strobe-statement.org.
